# The Prevention of Crime

**Published:** 1921-03-12

**Authors:** 


### THE PUBLIC WELL-BEING.

### The Prevention of Crime.

### DEFECTS OF THE PRESENT SYSTEM.

A valuable contribution has just been made by Dr. W. A. Potts, psychological expert to the Birmingham justices, to the discussion concerning crime and its prevention. It is evident, from the repeated observations of both magistrates and judges, that a large number of those who have to administer justice are growing to realise that all methods of dealing with crime are bad which tend to make a man a criminal when he might be saved by proper treatment, and that even the Children's Courts, though they serve within limits a useful purpose, are quite inadequately used for meeting the problem. It must be a common experience for any man whose business it is to attend courts of law to witness the despair of judges in having to re-sentence batch after batch of old criminals, who have spent most of their lives in and out of gaol, without any hope of safeguarding the public or reclaiming the prisoners; and the bewilderment of magistrates in dealing with young first offenders, whose mentality and lives they know nothing about, and with regard to whom they instinctively recognise that a form of punishment and a moral appeal are equally ineffective.

There need be no false sentiment about this matter. Crime ought not to be allowed to go unpunished, and the law-abiding citizen is entitled to the protection of the law by the fear which it should inspire in law-breakers, and which up to a point it does so inspire. But we cannot help thinking that the methods of the law in dealing with wrongdoers are rigid and unimaginative, and that the psychology of crime has been neglected until quite recently. Dr. Potts lays his finger on the spot in pointing out that many cases of failure and unsatisfactory conduct may be largely explained by conditions which have been almost entirely beyond the control of the individual, and even quite unrecognised by him. He does not, of course, attempt to explain away every case of criminality on these grounds. It must clearly be admitted that a certain number of criminals take the bad course because it offers them an easier and preferable mode of life. There are a large number of criminals who are never caught by the police, and the experiences of the expert criminologist are derived in the main from the less successful criminals, who as a class must be inferior in mental and physical ability to the police. The two important facts which Dr. Potts asserts are that the present method of dealing with crime is a failure, and that the habitual criminal always starts at an early age.

In support of his first contention, he points out that 62 per cent. of the persons convicted in England have been convicted before; that, of these 7 per cent. actually had twenty previous convictions; while, of the most serious convictions at the Assizes and Quarter Sessions, 70 per cent. have been previously convicted, which at least goes to prove that the existing methods do not to any large extent protect society. As to the age at which criminality develops, Dr. Charles Goring, who gained his experience inside a convict prison, regarded nineteen as the age, but the evidence now available suggests that it is much earlier. We are inclined to agree with Dr. Potts' view that one of the most serious mistakes of which magistrates are guilty in this country is to make it an almost invariable practice to dismiss the first offender. These cases should not be dismissed, because a first appearance by no means indicates that it is the first time the offender has gone wrong. To dismiss them, therefore, is not to dispose of them. Dr. Potts believes that the habitual criminal begins at ten years of age, and in some cases as early as seven years—a belief supported by the personal history of notorious criminals recently before the courts. Crime is a form of conduct, and the organ of conduct is the mind. Therefore in any attempt to deal rationally with crime it is necessary to study the mind of the individual who commits crime. One of the commonest causes of crime is mental defect, and the extent to which it is a definite cause is scarcely realised either by the general public or those who administer the law. This brings us to the question of psychological tests, which are still in their infancy; but it is becoming clear that, while criminality will never be destroyed, crime will be greatly diminished through investigation and reform along these lines, by getting at the criminal when he is young; for the task of controlling the old and hardened criminal will always be a hopeless one. Apart from the mentally defective, there are two other groups of delinquents classified by Dr. Potts: (1) Those whose wrongdoing can be explained to a considerable extent either by physical ill-health, unsatisfactory training in a wrong occupation, or bad companions; and (2) those for whom some special form of examination is necessary in order to find out what is the fundamental cause for their going wrong.

Let us take a few recent cases in the courts to show that they were essentially cases which ought to have been examined and treated in accordance with what was found to be in their condition.

A boy convicted for stealing was fourteen years of age. He had been stealing money in London, and then went to Dover, stole various things, bought a revolver, stole a bicycle and a pair of field-glasses. When put in the cells he very nearly succeeded in cutting a way out.

A woman convicted of theft was stated to have been stealing since she was eight years of age. Two thousand articles were in court which she had stolen from shopkeepers. She said she could not help it.

Another case was that of a boy who was arrested for fighting his brother, six years old, and pushing him into the canal, and was simply discharged by the court. The unusual appear-

**THE PUBLIC WELL-BEING**—(*continued*).

ance of the photographs published of him at once gave an impression of abnormality.

Reform is needed enabling all such cases to be treated other than by an inflexible code, although the Probation Act has already conferred on magistrates considerable and valuable powers of discretion and treatment, the extent of which some of them do not seem to understand. But that there is a better way of minimising juvenile crime than by process of treatment under the law is shown in the revelation, made possible by searching topographical tests, that such crime is practically non-existent where children in towns live close to a large public park. That is really a remarkable disclosure, the importance of which ought to be impressed on the Ministry of Health, for it demonstrates beyond dispute that not only the physical health of the people, but its moral well-being, is bound up in the provision of spacious town-planning schemes and the opening up of playing-grounds and wide open spaces in every town and city in the kingdom.

